# A cost minimization analysis of the implementation of the international lung screening trial in Catalonia (Spain)

**DOI:** 10.1186/s12913-025-13008-w

**Published:** 2025-07-30

**Authors:** Antoni Rosell, Sonia Baeza, Rocío Mouriño, Maria Saigí, Marta Munné, Pedro López de Castro, Jordi Bechini, Oriol Estrada, Jordi Ara, Laura Ricou, Francesc López-Seguí

**Affiliations:** 1 Barcelona Respiratory Network (BRN), Thorax Institute, Germans Trias i Pujol University Hospital, Autonomous University of Barcelona (UAB), Germans Trias i Pujol Research Institute (IGTP), Barcelona, Spain; 2 Germans Trias i Pujol University Hospital, Department of Medicine, Autonomous University of Barcelona (UAB), Germans Trias i Pujol Research Institute (IGTP), Barcelona, Spain; 3 Centre for Research in Health Economics (CRES), Pompeu Fabra University, Barcelona, Spain; 4 Catalan Institute of Oncology, Badalona, Barcelona, Spain; 5 Thoracic Surgery Service, Germans Trias i Pujol University Hospital, Badalona, Barcelona, Spain; 6 Directorate of Healthcare Strategy, Northern Metropolitan Territorial Management, Catalan Institute of Health, Barcelona, Spain; 7 Northern Metropolitan Territorial Management, Catalan Institute of Health, Barcelona, Spain

**Keywords:** Cost analysis, Early stage, Health economics, Lung cancer, Screening program

## Abstract

**Background:**

NLST and NELSON trial showed that lung cancer mortality can be reduced by 20–24% using low-dose computed tomography screening, due to an increase in early-stage diagnoses.

**Research question:**

How much lung cancer-related direct costs may be reduced using low-dose computed tomography screening based on the ILST-protocol in a public healthcare system?

**Methods:**

Cost analysis of lung cancer screening vs. usual care in the framework of the retail price of the Catalan public healthcare system. The lung cancer screening group included costs of screening (ILST-protocol), treatment cost according to weighted average distribution of TNM staging in the NLST and NELSON trials, lung cancer detection rate and smoking-cessation intervention. The usual care group included treatment costs based on distribution of TNM staging registered in the Spanish index hospital.

**Results:**

In the usual care group, treatment costs were €91,959. In 5-year of lung cancer screening program, the average expected costs per subject were €1,342 (range €1,054 − 1,832) for screening and €32,431 for treatment, with an expected reduction of €952 based on an average cancer detection rate of 1.6%. The decrease in cost resulting from the stage shift offsets 70.6% of the costs of the screening program.

**Conclusions:**

The decrease in direct costs associated with lung cancer treatment due to a stage shift resulting from LCS of high-risk populations compensates for a substantial part of the LCS program costs.

**Trial registration:**

Retrospectively registered.

## Background

Lung cancer is the leading cause of cancer death worldwide. In 2020, 2,206,771 new cases have occurred and caused 1,796,144 deaths [1]. Also in 2020, 4,509 cases and 3,548 deaths were registered in Catalonia (8 million inhabitants), Spain [2]. Among patients with non-small cell lung cancer (NSCLC), the 5-year survival rate is 26% for all stages and below 3% for stage 4 disease. Unfortunately, less than 20% of lung cancers are detected in localized tumor stages when it is most treatable [3–5]. In 1999, the Early Lung Cancer Action Program (ELCAP) study showed that screening with low-dose computed tomography (LDCT) improved early lung cancer detection [6]. In 2011, the U.S. National Lung Screening Trial (NLST) reported a 20% reduction in lung cancer-specific mortality using LDCT compared with chest radiography (CXR) screening in a high-risk population and a 6.7% overall mortality reduction [7]. In 2020, the Dutch-Belgian lung-cancer screening trial (NELSON study) in high-risk male participants showed a cumulative rate ratio for death of 0.76 at 10 years in the LDCT screening group as compared with no screening, although the difference was not deemed statistically significant for women [8]. Additionally, the Multicentric Italian Lung Detection (MILD) trial showed a 39% reduced risk of LC mortality at 10 years [2]. At the beginning of 2022, the European Parliament resolution recognized the evidence that proves the positive effect of targeted lung cancer screening on mortality [9], and a recent resolution from the European Commission Directorate-General for Health and Food Safety (COM 2022/474) has included lung cancer screening as a recommendation for the first time [10]. Once the clinical benefits of the LDCT screening on the high-risk population have been demonstrated, there are currently multiple initiatives focused on improving its cost-effectiveness, implementation in populations at risk and smoking cessation interventions [11].

The introduction of immunotherapy and targeted therapies in the management of advanced stages of lung cancer have shown considerable impact on overall survival (from 11.0 to 17.8% for the period between 2011 and 2014) in the subset of responders [12, 13]. These important changes are achieved at a high economical cost but could be offset by identifying early-stage tumours and treating them with the current standards of surgery or stereotactic body radiotherapy (SBRT). Lung cancer screening could avoid the expensive and less effective current treatments in use for advanced stage disease [14].

Cost-effectiveness analyses of lung cancer screening using LDCT is sensitive to different key model parameters (e.g., incidence, sensitivity and specificity of the tests, selection criteria, definition of high-risk population, utility values, etc.) showing inconclusive data and some uncertainty [15–17]. In the Pan-Canadian Early Detection Lung Cancer Study, the average cost to screen individuals with a high risk for developing lung cancer using LDCT and the average initial cost of curative intent treatment were found to be lower than the average per-person cost of treating advanced stage lung cancer [18]. Late treatment rarely results in a cure. In Catalonia, a cost-effectiveness analysis of different smoking cessation approaches concluded that the most cost-effective strategy would be to implement intensive smoking cessation interventions at ages 35–45, combined with LDCT screening every three years between the ages of 55 and 65 [19]. Another study in the same region showed that surgical treatment for early-stage lung cancer is cheaper and offered better outcome than advanced stage medical treatment and that the intervention would save money between 3 and 6 years after its launch [20]. Other studies have shown similar results [21].

In December 2020, an experimental lung cancer LDCT screening (LCS) program was implemented in the Northern Metropolitan Area of Barcelona through the participation of Germans Trias i Pujol University Hospital (HUGTP) in the international consortium of the International Lung Screen Trial (ILST), in which two patient selection strategies (PLCOm2012 vs. USPSTF 2013) and two protocols to classify the detected pulmonary nodules (PanCan vs. LUNG-RADS) were compared [22]. In this context, the aim of the present study was to perform a direct cost analysis comparing the reduction of overall lung cancer treatment expenses arising from stage shift due to screening, with the costs associated with usual care (no screening) in patients diagnosed with NSCLC in the framework of the public national health system in Catalonia (Spain).

## Methods

### Study design and objective

A cost analysis study was designed from the perspective of the Catalan Ministry of Health, the public health system that offers universal, free healthcare in Catalonia.

**Fig. 1:**
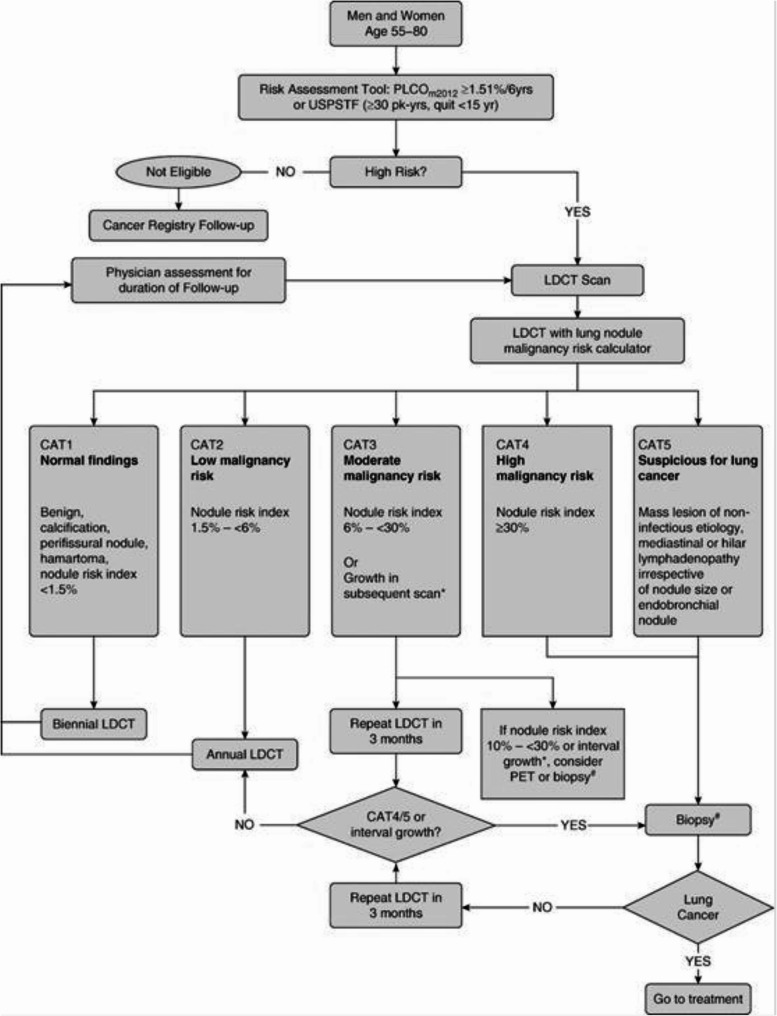
Lung nodule management protocol [22]. *Growth in subsequent scan is defined as: 0.1.5 mm in mean diameter or solid core of semi-solid nodule > 6 mm. # = Consider biopsy after appropriate clinical assessment. CAT = computed tomography; LDCT = low-dose computed tomography; PET = positron emission tomography; PLCO = prostate, lung, colorectal and ovarian; USPSTF = U.S. Preventive Services Task Force. Reprinted with permission of the American Thoracic Society. Copyright © 2022 American Thoracic Society. All rights reserved

The objective of the study was to compare the anticipated costs of diagnosis, treatment (surgery, radiotherapy and drugs) and follow-up associated with two approaches to the NSCLC diagnosis and care: the lung cancer LDCT screening according to the ILST protocol [22] versus the diagnosis in the usual care (Fig. 1). Usual care treatment was defined as the application of clinical practice guidelines developed by the Spanish Society of Medical Oncology for the treatment of NSCLC [23]. In both alternatives, all lifetime costs (up to healing or death) were taken into account. The costs, expressed in euros (2021), were calculated based on the standard retail prices of the health resources contractor of the Catalan Ministry of Health [24]. As a cross-sectional study, we did not include QALY (Quality-Adjusted Life Year) in the analysis.

### Estimated costs

The anticipated costs of the LCS program were calculated as the sum of the prices of radiological tests, medical visits, endoscopic or computed tomography-guided biopsies, laboratory tests, lung function tests and positron emission tomography (PET-CT), which were expected to be performed over a duration of 5 years according to the ILST protocol [22], considering 300 participants and assuming the following patients’ expected distribution: CAT1 (normal findings) 65.7%, CAT2 (low malignancy risk) 17.5%, CAT3 (moderate malignancy risk) 13.1%, and CAT4/5 (high malignancy risk/suspicious for lung cancer) 3.6% [25]. The costs associated with smoking cessation treatment (9 visits per patient) were also included, assuming that it will be necessary for 50% of participants, based on the patients enrolled at the LCS program during 2021. The costs of nicotine replacement treatment are not included in this study because they are not funded by the Catalan Healthcare Service.

Diagnostic procedures including bronchoscopy, staging CT, PET-CT, brain nuclear magnetic resonance and follow-up visits were recorded as per local practice guidelines (usual care). Treatment-related costs were calculated based on the main treatment schedules’ retail prices, administered for each stage of the disease and according to the institutional activity registry, which includes surgery, chemotherapy, radiotherapy, immunotherapy and targeted therapy. Total costs were estimated adjusted by overall survival based on the 8th edition of the American Joint Committee on Cancer (AJCC) staging manual (TNM) [26]. Although the public health system covers the whole treatment, the model did not include complications and second-line treatments; other, minor costs were not considered.

The stages distribution for usual care (stage I 14%, stage II 6%, stage III 12%, stage IV 68%) corresponded to data observed in 388 patients diagnosed with NSCLC at the Catalan Institute of Oncology, an institution that includes a network of public hospitals that in 2022 covered 40% of the population of Catalonia. The stages distribution contemplated for the screening alternative was estimated from the weighted average of the detections in the NELSON [8] and NLST [7] studies, eliminating the missing values (stage I 63%, stage II 8%, stage III 17%, stage IV 12%).

The total anticipated costs derived from the diagnosis, treatment and follow-up of the different lung cancer stages identified in each alternative were compared in order to assess the economic impact of early detection of the disease, using a 5-years average cancer detection rate (CDR) of 1.6%. As we present the first-round data, the average CDR corresponding to the 5 years program was taken from the Lahey program, which has a baseline CT CDR of 2%, identical to our site [27].

## Results

### Screening program

In the LCS program, total costs estimated for 300 patients expected to be managed in our centre amounted to €409,917. As shown in Table 1, the most substantial anticipated expenditure corresponded to LDCT tests (23.2%) followed by PET-CT (19.4%) and smoking cessation visits (16.7%), whereas biopsies (2.4%) and laboratory tests (0.4%) accounted for a minor part of anticipated costs. The average cost per participant for the 5-year duration of the LCS program was €1,349.72. Differences in cost of screening due to variations in the management of the lung nodule as established in the ILST protocol for each of the CAT (based on PanCan lung nodule risk-based protocol) categories are shown in Table 2. The cost per participant increased from €1,054 for CAT1 to €1,832 for CAT4/5 (a 74% increase).

**Table 1:** Anticipated costs of the LDCT screening program following the ILST protocol [22] in 300 patients

Resource	Quantity	Cost/unit, €	Total cost, €	Percentage of total cost
Initial medical visit	300	170	51,000	12.6
Lung function tests	300	164	49,200	12.2
Laboratory tests	300	5	1,500	0.4
LDCT	853	110	93,814	23.2
Follow-up medical visit	671	80	53,695	13.3
Smoking cessation visit	1,350	50	67,500	16,7
Lung nodule biopsy + bronchoscopy/lung CNB	39	242.14	9,552	2.4
PET-CT	79	997	78,656	19.4
Total cost of the screening program			409,917	100
Mean cost per participant over the 5 years			1,342.72	

*LDCT* low-dose computed tomography, *ILST* International Lung Screening Trial, *NSCLC* non-small cell lung cancer, *PET-CT* positron emission tomography/computed tomography, *CNB* core needle biopsy

**Table 2:** Anticipated costs of the screening according to CAT category and lung nodules following the ILST-protocol [22]

CAT	Management of lung Nodule^a^	Cost per participant, €
1	1 LDCT every 24 months (x 2 LDCT in 5 years)	1,054
2	1 annual LDCT during 2 years for solid nodules (x 2 LDCT)	1.054
	1 annual LDCT for 5 years for subsolid nodules (x 4 LDCT)	1,434
3	1 LDCT at 3 months, 1 PET-CT (± 50% of cases) + 1 nodule biopsy lung cancer (± 25% of cases) if the risk of malignancy is between 10% and 30% or there is growth of the lung nodule	1,483
4/5	1 PET-CT + 1 biopsy of the lung nodule (± 50% of cases)	1,832

^a^Lung nodule management at the start of screening includes for all participants the same visits and screening tests, which include 1 initial medical examination, 1 LDCT, 1 blood analysis with haemogram, biochemistry and coagulation tests, 1 lung function test and 4.5 smoking cessation treatment visits per year for 2 years (calculated considering that 50% require smoking cessation, and each of these 9 visits in total)

*CAT* computed tomography, *ILST* International Lung Screening Trial, *LDCT* low-dose computed tomography, *PET-CT* positron emission tomography/computed tomography

### Treatment costs of the NSCLC patient

There were relevant differences in the total cost of diagnosis, treatment and follow-up depending on the TNM stage in which the disease is detected, with 12 times higher costs for stage IV than those associated with stage I (Table 3).

**Table 3:** Anticipated costs of the usual care for the patient with NSCLC according to TNM stage

Stage	Average Cost per patient, €			
	Diagnosis	Treatment	Follow-up	Total cost
I	904	7,664	1,618	10,186
II	904	8,560	1,259	10,723
III	1,854	56,835	1,083	59,772
IV	1,566	119,754	1,023	122,343

*NSCLC* non-small cell lung cancer

### Stage shift and cost reduction

The differential costs arising from stage shift are detailed in Table 4. The average expected cost of diagnosis, treatment, and follow-up for lung cancer detected in LCS program was €32,431 as compared with €91,959 in the usual care. In the LCS program strategy, costs were mostly associated with stages I, II, and III (positive differential costs) because of a higher number of cases detected in the initial stages. However, the high cost associated with stage IV and the high proportion of these stages in the usual care program offset the expenditure on initial stage treatments in the screening program approach. Consequently, the differential cost per participant diagnosed with lung cancer was estimated at €59,528.

**Table 4:** Anticipated costs of diagnosis, treatment, and follow-up by participant in both strategies

Stage	Cost, €	Screening program with LDCT, € (%)	Usual care, € (%)	Differential due to stage shift, ∆€
I	10,186	6, 409 (62.9)	1,470 (14.4)	4,939
II	10,723	831 (7,7)	636 (5.9)	196
III	59,772	10,208 (17.1)	7,240 (12.1)	2,968
IV	122,343	14,982 (12.2)	82,613 (67.5)	−67,631
Total expected mean cost, €		32,431	91,959	−59,528

*LDCT* low-dose computed tomography

For a 5-year average CDR of 1.6%, the expected cost reduction per LCS for each participant was €952. Taking the expected cost reduction into account, the screening program in discounted cost was €397 (€1,349-€952), or €119.180 for 300 participants. The decrease in costs of diagnosis, treatment and follow-up resulting from stage shift offset 70.6% of the costs of the LCS program. Assuming a 1% cancer detection rate, decrease in costs of diagnosis, treatment and follow-up resulting from stage shift offsets 44% of the costs of the screening program. With a 2.2% cancer detection rate, 97% of the total costs would be offset (Table 5). In all the cases studied, the cost reduction resulting from the stage shift due to the LCS program offset a substantial part of the program costs.

**Table 5:** Costs of the screening program associated with three detection rates

Costs	Detection rate		
	1%	1.6%	2.2%
Screened cost per participant (A)	€1,349		
Expected value of the decrease in cost associated with stage shift per participant (B)	€595	€952	€1,309
‘Real’ cost of screening per participant (A-B)	€754	€397	€40
Total costs savings (B x 100/A)	44%	70%	97%

## Discussion

The stage of lung cancer diagnosis is a major determinant of lung cancer prognosis. A late diagnosis is the main contributing factor to the high frequency of individuals with advanced disease at presentation who are unlikely to benefit from curative treatment and accounts for the high lung cancer-specific mortality rates. In addition to the high mortality, the costs of non-curative treatment options, including new therapeutic targets, are very high and continue to rise. LCS programs are therefore essential to find lung cancers earlier and diagnose and treat more patients at early stages.

This study highlights the importance of the participant selection criteria and recruitment effectiveness in the potential savings of a LCS program. If only a prevalence of 1% is found, then the savings covers less than half of the direct costs of screening, while if a 2.2% cancer detection rate is achieved, then savings almost cover costs (Table 5). In relation to this, risk models appear to be more efficient than categorical age and smoking criteria, as shown in the interim results of ILST study in which USPSTF2013 categorical and the PLCOm2012 risk prediction model were prospectively compared, the latter being more efficient with a cancer sensitivity improvement of 15.8% [28]. Another approach to achieve better recruitment is screening low socioeconomic status population. Results of the UK Lung Cancer Screening trial [29], in which LDCT screening was evaluated in a large population sample of people aged 50–70 years, showed that higher socioeconomic status correlated positively with response, but inversely with risk. The proportion of individuals with high-risk of developing lung cancer (5% or greater over next 5 years– LLP risk prediction model) decreased with higher economic status, ranging from 17.7% in the most deprived quintile to 8.0% in the least-deprived quintile (*p* < 0.001). Also, a cost-effective screening program should include a significant proportion of women as the benefit has proved to be greater. In the NELSON study women achieved a 59% reduction in lung cancer-specific mortality as compared to 24% for men at 8 years of follow-up (although this difference decreases after 10 years) and 27% reduction for women in the NLST as compared to 8% for men [8, 30]. Recently, Cressman et al. have published with ILST data that the PLCOm2012 risk model compared USPSTF-2013 criteria saved costs, increased QALYs and decreased disparities in access to screening (mainly socioeconomic and sex-based differences) that support the use of risk models [31]. Although the categorical criteria have been modified in USPSTF2021 by lowering age and tobacco consumption (55 to 50 years and 30 to 20 pack-years) to reduce disparities these approach does not appear to be more cost-effective than risk models [32].

This study also differs from previous ones as the cost of smoking cessation is included. The total cost of smoking cessation visits represents 16.7% of the total cost of the LCS program. Including smoking cessation interventions within LCS program has additional benefits of improving tobacco-related health outcomes, such as chronic obstructive pulmonary disease, heart disease and other malignancies. This impact has not been quantified yet, but it is expected that it will contribute to cost-effectiveness of the LCS program [19]. In addition, it should be noted that while NELSON and NLST monitored normal CT results every year and a half and year respectively, in the ILST protocol this was done every two years. On the one hand, this reduces the costs of screening, but reduces its potential for detecting early stages.

The evidence from this research is consistent with previous studies [14] showing that, in high-risk populations, the average costs of LCS using LDCT plus the average initial cost of treatment with curative intent are less than the average cost per NSCLC patient with advanced stage in the usual care approach.

This study has some limitations. First, the cost analysis was based on a combination of our own data and estimated distribution of lung cancer obtained in a LCS program from the weighted average of two main published studies [7, 8]. Second, although the financial cost of lung cancer is high, the cost range in our study has been restricted to the healthcare system’s perspective, which according to some studies may account for less than 25% of the total costs attributable to the disease [33]. It does not consider a set of other potentially relevant costs, such as mortality and morbidity losses informal costs [34], costs related to primary care [35] or cancer drugs prices [36]. In addition, the expected positive effects of the smoking cessation program have not been taken into account, which implies that the results presented by this study could be even more favorable to screening. Third, adherence to the LCS program has been considered to be 100%, while published data varies between 50% and 90% so that our cost savings estimates are likely optimistic [37]. Fourth, as we only have baseline results, we cannot provide our own average CDR for the 5-year program. For our model, we have used the average CDR from the Lahey program [27] (1.6%) as their baseline CT CDR is equal to ours (2%). Additionally, the economic analysis has been based on the tariffs and clinical protocols of a public health system, which might differ from those of the private sector. Finally, the screening program is supposed to recruit the vast majority of high-risk individuals. However, if its implementation is unfortunately low, the ultimate impact on screening costs could be unfavorable.

The present findings invite a reflection on the current global health policy management of lung cancer along time. Current policy seems to be focused on short-term costs, to the detriment of the implementation of more mid-long term cost-effective solutions.Apart from increasing the smoking prevention and cessation program, including the cost of screening into the current budget for lung cancer diagnosis and treatment, will result in a certain cost increase of the budget for an indeterminate period. If the implementation of the LCS program is rapid, the selection criteria used is highly cost-effective, and the recruitment manages to reach the entire at-risk population, but especially the most socioeconomically deprived population, the increased cost of screening will be offset by the reduced treatment costs for early-stage in a relatively short time.

Finally, the potential risks and harms of LDCT screening should be mentioned. The identification of indeterminate nodules implies the repetition of CT scans with the consequent costs and risk of radiation and, in some cases, invasive complementary scans are performed. In addition, the wait until diagnosis involves distress to patients during the screening process.

Research on LCS should continue to further optimize workflows so that they become even more cost-effective. In addition to the use of LDCT as a screening tool, other fields of research such as blood biomarkers focused on both screening participant selection and the management of detected lung nodules [38] or the introduction of radiomics [39–41] are being developed. Once these techniques are validated in clinical practice, further cost-effectiveness studies will be needed.

## Conclusions

In conclusion, the decrease in direct costs associated with lung cancer treatment due to a stage shift resulting from LCS of high-risk populations compensates for a substantial part of the LCS program costs. Since the economic impact of a LCS program depends mostly on the cancer detection rate achieved, crucial aspects include the choice of the most effective selection criteria, the implementation of a robust public health policy to promote smoking cessation, screening most of the high-risk population especially the highest risk individuals, and achieving adherence to follow-up annual and biennial screening.

## Data Availability

Study data are available from the corresponding author upon request.

## Abbreviations

AJCC: American Joint Committee on Cancer
CXR: Chest radiography
ELCAP: Early Lung Cancer Action Program
HUGTP: Germans Trias i Pujol University Hospital
NLST: National Lung Screening Trial
NSCLC: Non-small cell lung cancer
LDCT: Low-dose computed tomography
PET: Positron emission tomography
SBRT: Stereotactic body radiotherapy
SCLC: Small cell lung cancer
LCS: Lung cancer screening
CAT: PanCan lung nodule risk based protocol
CDR: Cancer detection rate
